# Comparison of various SYSADOA for the osteoarthritis treatment: an experimental study in rabbits

**DOI:** 10.1186/s12891-015-0572-8

**Published:** 2015-05-20

**Authors:** María Permuy, David Guede, Mónica López-Peña, Fernando Muñoz, José-Ramón Caeiro, Antonio González-Cantalapiedra

**Affiliations:** 1 Clinical Sciences Department, Veterinary Faculty, University of Santiago de Compostela, Lugo, Spain; 2 Trabeculae S.L, Ourense, Spain; 3 Cooperative Research Thematic Network in Ageing and Frailty (RETICEF), Carlos III Health Institute. Ministry of Economy and Competitiveness, Madrid, Spain; 4 Orthopaedic Surgery Service, USC University Hospital Complex, Santiago de Compostela, Spain

## Abstract

**Background:**

Osteoarthritis is thought to be the most prevalent chronic and disabling joint disease in animals and humans and its treatment is a major orthopaedic challenge because there is no ideal drug treatment to preserve joint structure and function, as well as to ameliorate the symptomatology of the disease. The aim of the present study was to assess, using histology, histomorphometry and micro-CT, the effects of the treatment with several drugs of the SYSADOA group and a bisphosphonate in a model of early osteoarthritis, comparing all the results obtained.

**Methods:**

Osteoarthritis was surgically induced by anterior cruciate ligament transection and partial meniscectomy on one knee of 56 rabbits; treatment was started three weeks after surgery and lasted 8 weeks; at the end of this period, the animals were sacrificed. Animals were divided into seven groups (8 animals each), one for each regimen of treatment (glucosamine sulfate, chondroitin sulfate, hyaluronic acid, diacerein, risedronate and a combination of risedronate and glucosamine) and one for the control (placebo-treated) group. Following sacrifice, femoral osteochondral cylinders and synovial membrane samples were obtained for their evaluation by qualitative and quantitative histology and micro-CT.

**Results:**

The model induced osteoarthritic changes in the knee joints and none of the treatments showed a significantly better efficacy over the others. Regarding cartilage thickness and volume, all the treatments achieved scores halfway between control groups, without statistical differences. Regarding the synovial membrane, diacerein and risedronate showed the best anti-inflammatory profile, whereas glucosamine and chondroitin did not present any effect standing the hyaluronic acid results between the others. Regarding the subchondral bone, there were no differences in thickness or volume, but risedronate, diacerein and hyaluronic acid seemed to have considerably modified the orientation of the trabecular lattice.

**Conclusions:**

Out of the different drugs evaluated in the study for OA treatment, diacerein and risedronate showed, in all the parameters measured, a better profile of effectiveness; hyaluronic acid ameliorated cartilage swelling and promoted bone formation, but with a poor synovial effect; and finally, chondroitin and glucosamine sulfate prevented cartilage swelling in a similar way to the others, but had no effect on cartilage surface, synovial membrane or subchondral bone.

## Background

Osteoarthritis (OA), the most common form of arthritis [1], is a heterogeneous chronic disease involving all tissues of the synovial joint, including cartilage, subchondral bone, menisci and peri-articular soft tissues. Risk factors include advanced age, genetic predisposition, obesity, joint injury and abnormal joint loading [2]. It is now accepted that the disease etiology includes various mechanical, biochemical and genetic factors, as well as molecular and enzymatic feedback loops, ultimately leading to the onset and progression of the disease [3]. Osteoarthritic changes are primarily the result of a disturbance in the remodelling process of joint tissues resulting from the failure of cells to maintain a homeostatic balance between synthesis and degradation; as the disease advances, the catabolic process exceeds the anabolic one, leading to progressive joint tissue lesions.

The pharmacological treatment of OA includes different drugs that can be classified on the basis of their mode of action. The most used options are focused on pain relief and improvement of joint function, including analgesics (such as acetaminophen) and non-steroidal anti-inflammatory drugs (NSAIDs); unfortunately, their use in chronic treatments has been limited by their deleterious side effects. Findings reported in the 1990’s about the metabolic activity of cartilage and subchondral bone led to the proposal of chondroprotective substances with the ability to provide symptomatic relief by targeting the underlying pathology of OA and with less secondary effects; such agents were classified as symptomatic slow-acting drugs for OA (SYSADOAs), including cartilaginous matrix precursors (glucosamine, chondroitin sulfate and hyaluronic acid) and cytokine modulators (diacerein) [4], as well as other types of drugs like bisphosphonates. They are expected to delay, stabilize or reverse the pathological changes in osteoarthritic joints, limiting the progression of the disease [5].

Glucosamine, an endogenous amino monosaccharide synthesized by chondrocytes, is the basic precursor of the structure of glycosaminoglycans and subsequently of aggrecan and other proteoglycans present in cartilage, which are part of the extracellular matrix. There are several available glucosamine preparations (e.g. sulfate or hydrochloride) that showed different clinical results, more favorable being those for glucosamine sulfate than for hydrochloride [6]. Glucosamine sulfate demonstrated its efficacy and clinical relevance in clinical trials [7] and animal models [8], helping to restore the proteoglycan matrix of the cartilage, protecting damaged cartilage from metabolic impairment [9] and showing a mild anti-inflammatory activity [10].

Chondroitin sulfate belongs to the class of natural glycosaminoglycans and is an unbranched complex polysaccharide consisting of a repeating disaccharide structure of glucuronic acid and N-acetyl-D-galactosamine [11,12]. It is a major component of the extracellular matrix (ECM) where it constitutes an essential component of proteoglycans. Its possible usefulness for OA treatment was demonstrated in animal studies [13], as well as in clinical trials [14,15].

Hyaluronic acid is a large, viscoelastic, linear polysaccharide found in the synovial fluid and cartilage. It is a macromolecule of several million Daltons consisting of repeating units of D-glucuronic acid and D-acetylglucosamine [16]. The main purpose of the viscosupplementation is to make up for the loss of viscoelasticity of the synovial fluid due to inflammation and to protect against cartilage degradation. Moreover, it performs biological activities in joint tissues, such as the inhibition of the production of metalloproteinases, chemokines and prostaglandins [17,18]. Its effects in OA were also demonstrated in animals [19,20] and in humans [21].

Diacerein, an anthraquinone derivative, is an oral anti-inflammatory, analgesic and antipyretic agent developed specifically for the treatment of OA. Rhein, the active metabolite, showed *in vitro* and *in vivo* the inhibition of the IL-1β -a pro-inflammatory cytokine, which stimulates the degradation process of cartilage and suppresses cartilage matrix synthesis [22], as well as the reduction of the collagenase production by chondrocytes, the reduction of the fibrinolytic activity in synovial fluid [23,24] and the stimulation of the production of cartilage growth factors [25], turning diacerein in an interesting option for the OA treatment supported by the findings in animal models [26] and in clinical trials [27].

Bisphosphonates (BPs), non-hydrolyzable analogues of inorganic pyrophosphate, have been approved for the treatment of pathologies with an increased bone turnover because they inhibit bone resorption by causing osteoclast apoptosis. There are many clinical and experimental evidences of other biological effects of BPs, which may act on other cells such as macrophages or chondrocytes. Bisphosphonates seem to inhibit matrix metalloproteinases [28] and have been reported to exert chondroprotective and analgesic effects in OA [29], even though the exact mechanisms still remain unclear. Risedronate is one of the most potent BPs and has demonstrated beneficial effects on OA progression. It was reported that the combination of risedronate and non-steroidal antiinflammatory drug therapy in the early OA stages preserve trabecular bone mass and reduce the impact of osteophytic bony adaptations and bone marrow lesions in a rat model [30]. Studies in guinea pigs suggest smaller cartilage lesions in risedronate-treated joints versus those of animals in control groups [31]. The efficacy of risedronate in the treatment of human OA was investigated in clinical trials with contradictory results; the BRISK (British Study of Risedronate in Structure and Symptoms of Knee OA) study [32] revealed clear trends towards improvement in joint structure and symptoms; however, the KOSTAR (Knee OA Structural Arthritis) [33] study showed that risedronate (compared with placebo) did not alter OA progression or improve signs and symptoms, although a reduction in biochemical markers of cartilage degradation was observed.

Despite the numerous *in vitro* studies, animal models and clinical trials supporting the beneficial effect of all these compounds for OA, and their potential disease modifying effect, there were some other studies in animals [34] and in humans [32] which questioned their benefits. This calls for further research into the potential usefulness of these therapies.

The aim of this study was to compare the effects of several osteoarthritis treatments on a rabbit surgical model, evaluating the magnitude of changes produced in the knee joint after eight weeks of treatment using histological, histomorphometric and tomographic (micro-CT) techniques.

## Methods

### Experimental animal model

Fifty-six healthy adult female New Zealand white rabbits (Granja San Bernardo, Navarra, Spain) of 6-7 months of age and mean weight 5 Kg were used in this study upon approval of the protocol by the Ethical Committee of the University of Santiago de Compostela (Reference number: AE-LU-002/09/FUN01/PAT.(06)E). Rabbits’ housing, daily monitoring and experimental procedures were conducted in the Animal Experimentation Facility of the University of Santiago de Compostela (Lugo, Spain) by accredited veterinarians, trained in laboratory animal science. All animal handling and experimentation were performed in accordance with Spanish and European Union regulations regarding care and use of research animals and this paper has been written in line with the ARRIVE statement [35].

**Figure 1:**
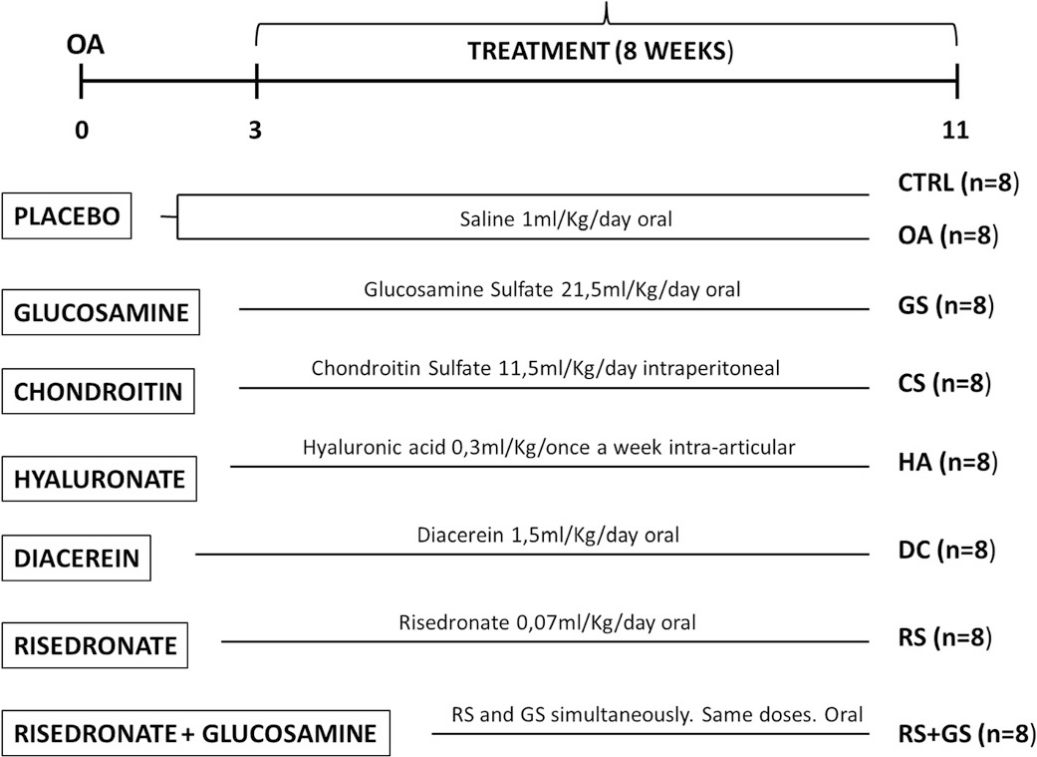
Experimental design. Groups of treatment.

The outline of the experiment, as well as the treatment schedule is presented in Figure 1.

After the quarantine, OA was induced by anterior cruciate ligament transection (ACLT) and partial medial meniscectomy on one knee of each animal randomly chosen. The anesthetic protocol and postoperative treatment used have been widely discussed in a previous study of our group [36]. The surgery was performed using a lateral parapatellar approach, medial dislocation of the patella and, with the knee in maximum flexion, anterior cruciate ligament and medial meniscus visualization and section. After surgery, the animals were housed in cages, allowed to perform normal activity and monitored daily to assess changes in general health.

Three weeks after the OA induction, animals were randomly divided for treatment into 7 groups of 8 animals each. Glucosamine sulfate, diacerein and risedronate (alone and combined with glucosamine) were orally administered diluted in normal saline (NaCl 0.9%) once a day, chondroitin sulfate was administered diluted in saline by intraperitoneal daily injection and hyaluronic acid diluted in phosphate buffered saline (PBS) was administered by intra-articular injection once a week. The placebo group was treated with saline orally once a day. All the dosages used in this study correspond to the human clinical doses recalculated for the rabbits’ mean weight. Animals were treated over a period of 8 weeks and then sacrificed for sample collection.

### Necropsy and preparation of histological samples

Rabbits were sacrificed by sodium pentobarbital overdose (100 mg/Kg IV, Dolethal, Vétoquinol especialidades veterinarias SA, Madrid, Spain) after sedation with ketamine (25 mg/Kg IM, Imalgène 1000, Merial, Toulouse, France). All knee joints were removed and immersed in 10% buffered formalin.

The protocol used for the preparation of the histological samples as well as their processing and evaluation were the same used in a recent previous study [36]. In summary: After dissecting the joint, two cores of bone and cartilage were obtained from the medial femoral condyle as well as a synovial membrane section. A cylinder was decalcified (Osteodec, Bio-Optica, Milano, Italy) and together with the articular capsule, paraffin embedded and stained with hematoxylin-eosin and safranin O-fast green (in the case of the bone and cartilage cylinder). These samples were used for evaluating OA articular changes according to the already published guidelines [37,38]. The assessed parameters and gradation of the changes observed in the structures are summarized in Table 1.

**Table 1:** **Gradation of the cartilage and synovial changes in non-calcified samples**

**CARTILAGE**	
**Severity of cartilage pathology**	
Normal volume, smooth Surface, intact	0
Surface undulations. Superficial zone fissures	1
Middle zone fissures and/or superficial erosions	2
Deep zone fissures and/or erosion through mid zone	3
Full thickness loss of cartilage	4
**Severity of chondrocyte pathology**	
Normal	0
Loos of superficial cells or relative increased density with occasional clusters	1
Small clusters (2–4 cells) predominate	2
Large clusters (up to 5 cells) predominate	3
Cell loss (necrosis/apoptosis) predominate	4
**Severity of proteoglycan pathology**	
Normal	0
Decreased proteoglycan in the superficial zone	1
Decreased proteoglycan into the mid zone	2
Decreased proteoglycan into the deep zone	3
Full depth	4
**Tidemark**	
Intact and distinct	0
Loss and/or duplication but distinct	1
Loss. Crossed by blood vessels	2
**SYNOVIAL MEMBRANE**	
**Lining cell characteristics**	
1-2 layers of cells	0
3-6 layers of cells	1
˃6 layers of cells	2
**Hyperplasia**	
No hyperplasia	0
Short villi	1
Finger-like hyperplasia	2
**Cell infiltration characteristics**	
No cellular infiltration	0
Mid to moderate inflammatory infiltration. Including small lymphoid follicles	1
Marked and diffuse inflammatory infiltration. Large lymphoid follicles.	2

Parameters evaluated in the gradation of histologic features in decalcified paraffin embedded samples [37].

The second cylinder was initially used to assess the 3D architecture by a high-resolution micro-CT system (SkyScan 1172, Bruker, Kontich, Belgium) using radiologic parameters which have already been published [36]; due to the different scanning conditions required to visualize bone and cartilage, each sample was scanned twice. Next, the samples were processed for undecalcified ground sections in accordance with the method described by Donath [39] and stained using the Lévai-Laczkó method [40].

With micro-CT scanning, standard indices of cancellous bone microstructure were determined in subchondral bone [41] including bone volumetric fraction (BV/TV; %), trabecular thickness (Tb.Th; μm) and separation (Tb.Sp; μm), trabecular number (Tb.N; 1/mm), trabecular bone pattern factor (Tb.Pf: 1/mm), structural model index (SMI) and degree of anisotropy (DA). In addition, volumetric bone mineral density (vBMD; mg/cm^3^) was determined by calibration against hydroxyapatite phantoms of known density. In cartilage, the non-calcified cartilage volume (nCg.V; mm^3^) and thickness (nCg.Th; μm) were measured three-dimensionally.

**Figure 2:**
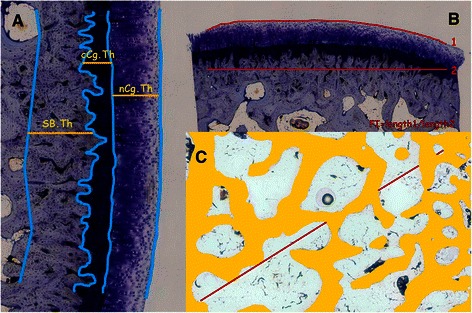
Representative images of the measurements made on undecalcified sections. **(A)** cartilage and subcondral bone cortical thickness (nCg.Th; cCg.Th; Cg.Th = nCg.Th + cCg.Th) SB.Th). **(B)**. Surface undulation (FI). **(C)**. Trabecular subchondral bone measurements in a ROI. Tb.A: % of trabecular bone in the ROI; Tb.Sp measured on the diagonal of the ROI (Tb.Sp = (1/Tb.N)-Tb.Th).

Next, the samples were used for quantitative histology applying morphometrical parameters [42], employing image analysis programs. The parameters evaluated were: subchondral bone cortical thickness (SB.Th; μm); cartilage thickness (Cg.Th; μm), measured separately for the non-calcified (nCg.Th; μm) and the calcified cartilage (cCg.Th; μm) using the tidemark as reference; surface cartilage undulations measured by the fibrillation index (FI); trabecular subchondral bone area (Tb.A; %) and separation (Tb.Sp; μm) measured in a region of interest of 4×2 mm located immediately after the subchondral cortical bone (Figure 2).

### Statistical approach

For the statistical evaluation, samples were divided into eight groups: CTRL (healthy knees of the placebo-treated group, which correspond to negative control), OA (operated knees of the placebo group, which are the positive control), GS (operated knees of the glucosamine sulfate-treated animals), CS (chondroitin sulfate), HA (hyaluronic acid), DC (diacerein), RS (risedronate alone) and RS + GS (combination of risedronate and glucosamine).

Results were expressed as mean ± standard deviation. The statistic comparison was made among all the groups (operated knees of treated animals), the positive and the negative controls. Normality of the data was assessed using the Shapiro-Wilk test. Levene’s test was used to assess the equality of variances of normal variables and the statistical comparison was performed using ANOVA. The post-hoc analysis was conducted by the Holm Sidak method. For non-normal variables, the statistical comparison was performed using the Kruskal-Wallis H test and the post-hoc analysis using Dunn’s test. All statistical analyses were performed using the commercially available software Sigma Plot 12.5 package (Systat software Inc., San Jose, CA, USA). Differences were considered significant when *p* < 0.05.

## Results

No changes in weight or general condition were observed during the experimental protocol and the drug administration was well tolerated by all the animals. The only outstanding fact was that animals treated with diacerein presented urine discoloration; however, no pathological changes in the kidneys or other organs were detected during necropsies. Three animals presented infectious changes in the operated knee so they were excluded from the analysis (the animals belonged to the OA, CS and HA groups).

The mean values and standard deviation of the different treatment groups are presented in Tables 2, 3 and 4.

**Table 2:** **Histological Analysis**

	CTRL	OA	GS	CS	HA	DC	RS	RS + GS
	mean ± SD	mean ± SD	mean ± SD	mean ± SD	mean ± SD	mean ± SD	mean ± SD	mean ± SD
**Cg pathol**	0.29 ± 0.49	1 ± 0.81	1.29 ± 0.76	1.29 ± 0.76	0.9 ± 0.55	0.5 ± 0.76	1 ± 0.82	0.38 ± 0.58
**Chondro p**	0.57 ± 0.78	1.21 ± 1.15	1.86 ± 1.46	1.14 ± 1.07	1.2 ± 1.10	1.81 ± 1.19	1 ± 0.82	0.94 ± 1.01
**PG pathol**	0.33 ± 0.81	1.29 ± 1.50	1.71 ± 1.7	1.5 ± 1.85	1.1 ± 1.14	1.56 ± 1.68	1.5 ± 1.76	2.38 ± 1.85
**Tidemark**	1.12 ± 0 + 83	0.67 ± 1.03	0.17 ± 0.41	0.67 ± 0.82	0.83 ± 0.41	0.88 ± 1.00	1 ± 0.58	0.79 ± 0.7
**Lining cells**	0 ± 0	1 ± 0.63	1 ± 0.93	0.83 ± 0.75	0 ± 0	0.29 ± 0.49	0.25 ± 0.46	0.31 ± 0.46
**Hyperplasia**	**0.13 ± 0.35**	**1.17 ± 0.75**	**1.5 ± 0.53**	**0.83 ± 0.75**	**1.25 ± 0.46**	**0.86 ± 0.69**	**0.5 ± 0.53**	**0.69 ± 0.59**
**Cell infiltrat**	0 ± 0	0.67 ± 0.52	0.69 ± 0.70	0.67 ± 0.52	0.75 ± 0.89	0.29 ± 0.49	0.38 ± 0.74	0.25 ± 0.46

Histology results of decalcified core samples and synovial membrane. Cg pathol: cartilage pathology; Chondro *p*: chondrocyte pathology; *PG* pathol: proteoglycan pathology; Tidemark: tidemark integrity; Cell infiltrate: cellular infiltration. Statistical significant parameters are marked in “bold text”.

**Table 3:** **Micro-CT analysis**

	CTRL	OA	GS	CS	HA	DC	RS	RS + GS
	mean ± SD	mean ± SD	mean ± SD	mean ± SD	mean ± SD	mean ± SD	mean ± SD	mean ± SD
**BV/TB**	48.46 ± 7.74	49.37 ± 4.38	44.74 ± 7.76	50.19 ± 50.29	52.86 ± 3.63	51.92 ± 4.99	51.71 ± 9.64	51.15 ± 2.82
**Tb.Th**	265.2 ± 32.62	288.75 ± 60.43	275.74 ± 82.04	286.19 ± 57.83	262.38 ± 31.06	281.18 ± 56.18	248.35 ± 18.48	255.3 ± 38.8
**Tb.Sp**	467.24 ± 32.6	423.12 ± 66.2	**478.00 ± 64.74**	431.02 ± 55.25	**353.7 ± 37.75**	433.27 ± 54.03	367.53 ± 61.9	385.09 ± 61.9
**Tb.N**	1.83 ± 0.20	1.76 ± 0.29	1.68 ± 0.28	1.77 ± 0.16	2.34 ± 0.23	1.89 ± 0.30	2.07 ± 0.3	2.03 ± 0.25
**Tb.Pf**	-3.27 ± 1.49	-3.24 ± 0.51	-3.15 ± 1.81	-3.76 ± 1.95	-4.17 ± 1.43	-4.01 ± 1.15	-4.09 ± 2.92	-4.31 ± 0.95
**SMI**	-0.66 ± 0.47	-0.72 ± 0.39	-0.51 ± 0.44	-0.88 ± 0.85	-0.85 ± 0.53	-0.96 ± 0.60	-0.87 ± 1.09	-0.8 ± 0.35
**DA**	**0.71 ± 0.06**	**0.69 ± 0.06**	**0.71 ± 0.05**	0.61 ± 0.06	**0.51 ± 0.04**	**0.57 ± 0.06**	**0.53 ± 0.10**	**0.52 ± 0.08**
**nCg.Th**	309.06 ± 63.0	494.8 ± 169.6	369.71 ± 113.74	388.04 ± 114.15	413.43 ± 55.2	359.75 ± 48.62	391.41 ± 68.7	359.77 ± 131.4
**nCg.V**	3.37 ± 0.78	5.69 ± 2.73	3.37 ± 1.49	3.68 ± 1.24	4.27 ± 1.56	3.71 ± 0.50	3.47 ± 1.11	3.29 ± 1.43
**vBMD**	505.9 ± 98.53	512.78 ± 52.52	471.32 ± 108.96	531.31 ± 68.58	562.51 ± 34.00	548.69 ± 58.95	549.24 ± 109.9	568.14 ± 42.44

Micro-CT results. *BV*/*TV*: bone volume/tissue volume; *Tb.Th*: trabecular thickness; *TB.Sp*: trabecular separation; *Tb.N*: trabecular number; *Tb.Pf*: trabecular pattern factor; *SMI*: structural model index; *DA*: degree of anisotropy; *nCg.Th*: non-calcified cartilage thickness; *nCg.V*: non-calcified cartilage volume; *vBMD*: volumetric bone mineral density. Statistical significant parameters are marked in “bold text”.

**Table 4:** **Histomorphometrical analysis**

	CTRL	OA	GS	CS	HA	DC	RS	RS + GS
	mean ± SD	mean ± SD	mean ± SD	mean ± SD	mean ± SD	mean ± SD	mean ± SD	mean ± SD
Tb.A	46.4 ± 5.4	47.9 ± 7.01	39.8 ± 8.8	44.5 ± 5.8	43.2 ± 6.1	45.5 ± 6.8	41.7 ± 8.1	38.9 ± 5.55
Tb.Sp	514.9 ± 235.2	437.8 ± 211.9	593.9 ± 214.4	503.3 ± 141.7	465.6 ± 139.5	590.1 ± 272.7	528.2 ± 272.7	731.26 ± 189.3
SB.Th	306.6 ± 76.5	251.6 ± 67.4	273.1 ± 109.96	288.1 ± 85.01	237.6 ± 50.5	268.2 ± 47.01	274.6 ± 96.7	229.7 ± 64.6
FI	1.1 ± 0.07	1.2 ± 0.14	1.3 ± 0.3	1.35 ± 0.6	1.14 ± 0.1	1.1 ± 0.05	1.06 ± 0.07	1.09 ± 0.13
Cg.Th	493.7 ± 80.7	620.1 ± 114.8	522.07 ± 120.1	506.4 ± 128.8	536.8 ± 98.9	571.1 ± 96.7	499.05 ± 56.4	555.7 ± 120.1
nCg.Th	338.6 ± 65.03	465.2 ± 88.9	364.6 ± 98.6	375.9 ± 113.3	411.2 ± 90.5	409.8 ± 82.6	320.3 ± 58.2	408.6 ± 88.7
cCg.Th	155.09 ± 24.9	167.75 ± 43.01	160.7 ± 25.8	132.6 ± 27.08	129.06 ± 18.4	161.3 ± 42.09	140.1 ± 35.8	156.01 ± 26.35

Histomorphometry scores of undecalcified samples. *TB*.A: trabecular area; *Tb.Sp*: trabecular separation; *SB.Th*: subchondral bone thickness; *FI*: fibrillation index; *Cg.Th*: total cartilage thickness; *nCg.Th*: non-calcified cartilage thickness; *cCg.Th*: calcified cartilage thickness.

**Figure 3:**
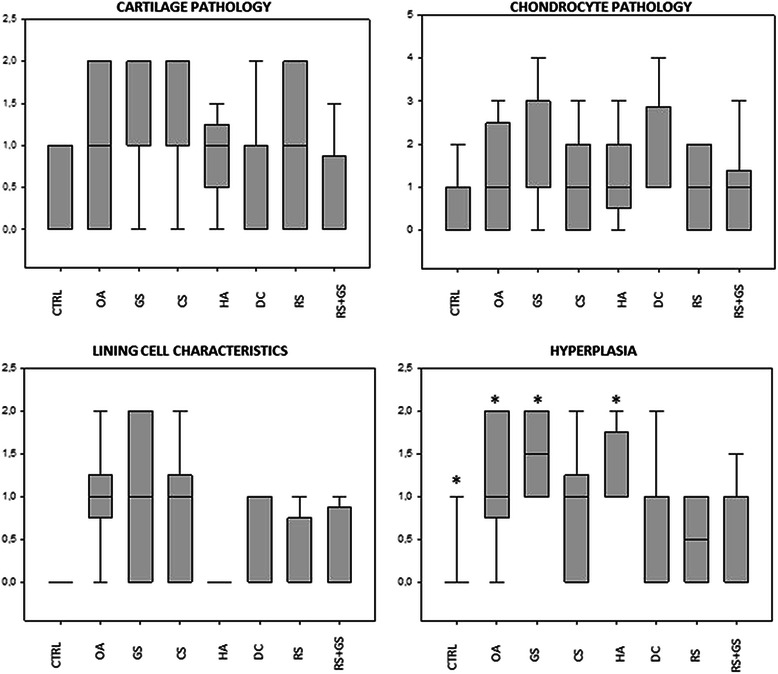
Box plot comparing the scores obtained by the different experimental groups for qualitative microscopic grading of cartilage and synovial alterations (decalcified samples). In hyperplasia of the synovial membrane the differences were shown between CTRL-GS, CTRL-HA and CTRL-OA and also between GS and RS. The rest of the parameters did not show differences among groups.

**Figure 4:**
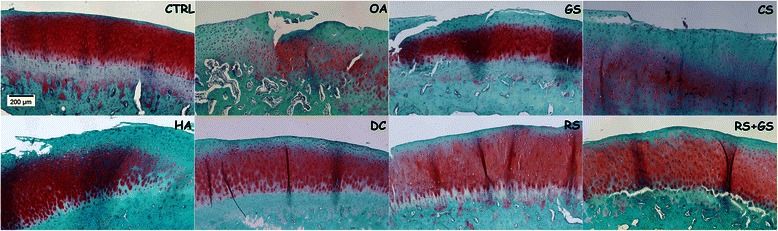
Decalcified cartilage samples. Representative histology images of the treatment groups in decalcified samples stained with Safranin-O. Magnification 10X. CTRL: healthy knees of the placebo-treated group; OA: osteoarthritic knees of the placebo group; GS: glucosamine sulfate; CS: chondroitin sulfate; HA: hyaluronic acid; DC: diacerein; RS: risedronate; RS + GS: risedronate + glucosamine.

Regarding the parameters measured in cartilage (Table 2; Figures 3 and 4), there were not statistical differences among groups; although the Kruskal Wallis comparison for the cartilage pathology was almost significant (p = 0.059) and the groups treated with diacerein (DC) and with the combination of risedronate and glucosamine (RS + GS) presented scores closer to the negative control (CTRL) than the others (Figure 3). The results for the different groups for the severity of cartilage pathology and chondrocyte pathology are graphically represented in a box plot in Figure 3.

In Figure 4 the groups corresponding to the positive control (OA), glucosamine (GS), chondroitin (CS) and hyaluronate (HA) showed loss of the superficial structure of the cartilage as well as loss of the red stain, especially in the superficial layers of the cartilage. The chondrocyte disorganisation, with cells not arranged in rows and the evident formation of clusters, could be observed with greater intensity in the images corresponding to the OA, GS and CS groups, whereas in DC, RS and RS + GS cells are better organized (similar to those in the negative control CTRL).

**Figure 5:**
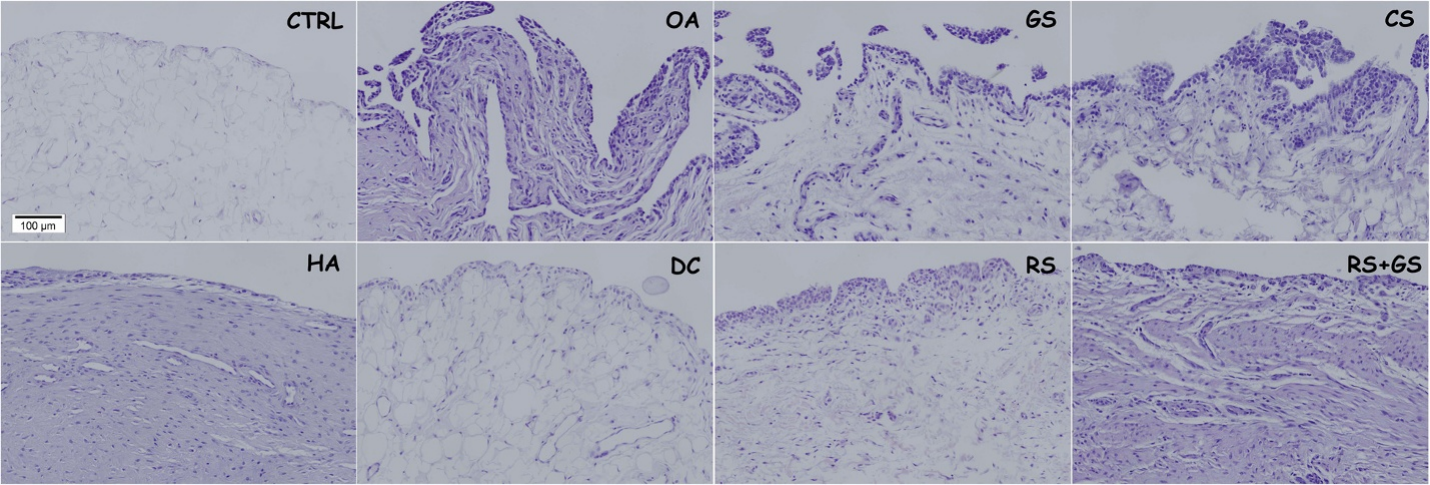
Synovial membrane. Representative histology images of the synovial membrane. Stained with H-E. Magnification 20X. CTRL: healthy knees of the placebo-treated group; OA: osteoarthritic knees of the placebo group; GS: glucosamine sulfate; CS: chondroitin sulfate; HA: hyaluronic acid; DC: diacerein; RS: risedronate; RS + GS: risedronate + glucosamine.

Regarding the synovial membrane (Table 2, Figures 3 and 5), the only measure that did not show any differences in the analysis of variance was cell infiltration; the others showed statistical differences, with p = 0.003 for the lining cells and p < 0.001 for hyperplasia (results plotted in Figure 3). When a multiple comparison method was used to isolate the groups, it was impossible to identify the differences in lining cells whereas in hyperplasia the differences were shown between CTRL- GS, CTRL-HA and CTRL-OA and also between GS and RS (Figure 3).

It is important to mention that the behaviur of the HA group in lining cell characteristics was the same as the one shown by the negative control (CTRL) with p = 1.

In the synovial membrane of the negative control group (CTRL) (Figure 5) the lining surface was thin, with one or two layers of cells, the surface was smooth, with no presence of short villi or finger-like hyperplasia, or of inflammatory infiltrations. The OA group (positive control) was in the opposite situation, with a thick lining surface, profuse hyperplasia, presence of diffuse inflammatory infiltration and a deposition of fibrous tissue below the intima tissue. The chondroitin sulfate (CS) and glucosamine sulfate (GS) showed an image similar to those of the OA group (with hyperplasia and more than two layers of superficial cells) but with less fibrous tissue in the subintima. Finally, in the other treatment groups (DC, HA, RS and RS + GS), the number of superficial cell layers tends to approach the one of controls, as well as hyperplasia, exhibiting less fibrous subintima tissue than the OA group but it is more marked than in the control group.

**Figure 6:**
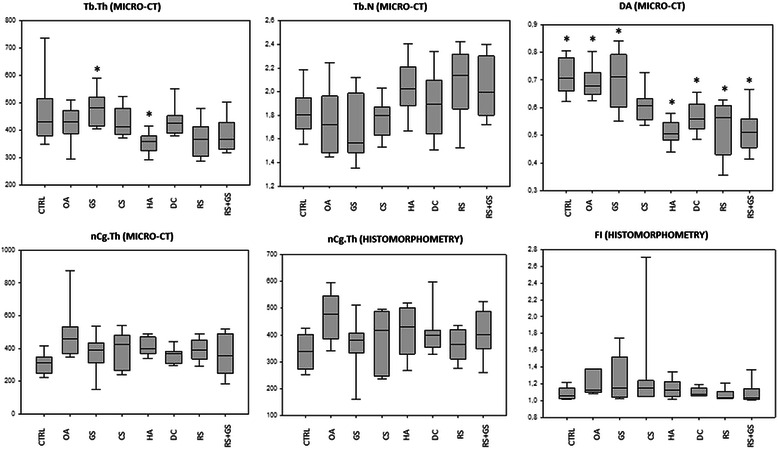
Box plot comparing the scores obtained by the different experimental groups for micro-CT and quantitative histomorphometry. In Tb.Sp measured by micro CT the groups that showed significance were GS vs. HA. In the parameter DA (degree of anisotropy) there were statistical differences between: CTRL-HA (p < 0.001), CTRL-DC (p = 0.003), CTRL-RS (p < 0.001), CTRL-RS + GS (p < 0.001), GS-HA (p < 0.001), GS-DC (p = 0.006), GS-RS (p < 0.001) and GS-RS + GS (p < 0.001); also between the OA-HA (p < 0.001), OA-DC (p = 0.018), OA-RS (p < 0.001) and OA-RS + GS (p < 0.001). In the rest of the parameters the differences were not significant.

Regarding the parameters measured by micro-CT (Table 3, Figure 6), the statistical differences were found in the trabecular number (Tb.N; p = 0.018), trabecular separation (Tb.Sp; p = 0.004) and the degree of anisotropy (DA; p < 0.001). When the groups were compared, Tb.N showed no significant differences while Tb.Sp showed them between GS and HA. With respect to the DA differences, they were found between CTRL-HA (p < 0.001), CTRL-DC (p = 0.003), CTRL-RS (p < 0.001), CTRL-RS + GS (p < 0.001), GS-HA (p < 0.001), GS-DC (p = 0.006), GS-RS (p < 0.001) and GS-RS + GS (p < 0.001); also between the OA-HA (p < 0.001), OA-DC (p = 0.018), OA-RS (p < 0.001) and OA-RS + GS (p < 0.001) groups. The results of these parameters are graphically represented in a box plot diagram in Figure 6.

The nCg.Th and nCg.V scores of all the treatment groups were halfway between the negative and the positive controls, but without statistical differences (Figure 6).

**Figure 7:**
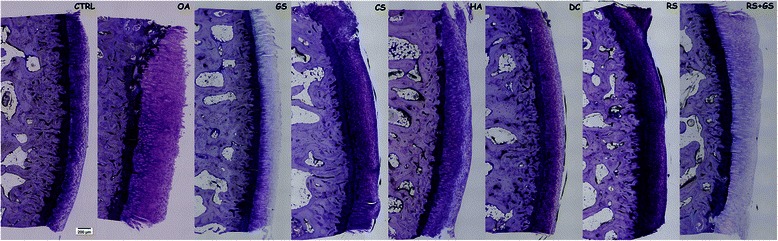
Calcified samples. Representative histology images of the histomorphometry samples. Non-decalcified. Levai-Laczkó staining. Magnification 10X. CTRL: healthy knees of the placebo-treated group; OA: osteoarthritic knees of the placebo group; GS: glucosamine sulfate; CS: chondroitin sulfate; HA: hyaluronic acid; DC: diacerein; RS: risedronate; RS + GS: risedronate + glucosamine.

Regarding histomorphometry (Table 4; Figures 6 and 7), there was no statistical significance among groups in any of the parameters measured, although the superficial fibrillation index (FI) showed a trend to approximate to the control group values. When the FI scores were observed in further detail, the DC, RS and RS + GS groups presented mean scores equal or even below those of the CTRL group while HA obtained scores halfway between CTRL and OA, whereas the other treatment groups (GS, CS) even higher values than OA (Figure 6). In the same way as in micro-CT, within the group of treated animals, the scores of the three measurements of cartilage thickness (total, non-calcified and calcified) were halfway between CTRL and OA, but they did not present any significant differences compared to any of them.

In the microscopic images of the calcified samples stained with Levai-Lazckó (Figure 7), despite small differences in the statistical analysis, several differences between groups were observed. Total cartilage (Cg.Th) of the OA group was thicker than the others, all the treatment groups reaching intermediate values between CTRL and OA. Another important finding that may be visualized in the images is that the OA group had more superficial fibrillation than CTRL (negative control). The images of the diacerein (DC), risedronate (RS) and risedronate plus glucosamine (RS + GS), as well as hyaluronic acid (HA) groups, showed less superficial irregularities similar to the negative control (CTRL), whereas glucosamine (GS) and chondroitin (CS) groups had more fibrillation. The distribution of chondrocytes in the cartilage layer, although the cell distribution and cluster formation were not evaluated in these calcified samples, was different too, showing more disorganization in the OA group than in the others. Finally, in the image of the OA group, the partial loss of the calcified cartilage (cCg.Th) could be observed, as well as in the glucosamine and chondroitin sulfate images, unlike in the others.

## Discussion

Animal models for OA, including rabbits, provide an important opportunity to solve the current controversy as to whether different drugs have a structure which leads to a detectable effect or not. Surgically induced models, resulting in joint instability, produce a gradual progression of degeneration which mimics the pathogenesis of the human traumatic OA [43,44]. Previously published data showed that most rabbits with ACLT develop cartilage degeneration and subchondral bone alterations [45] as soon as 8 weeks post-surgery. In the present study the OA was enough to produce detectable changes in several joint tissues, although it was still in an initial stage (changes are more evident in the placebo OA group). The articular cartilage is in a state of swelling, characteristic of the initial stage of the disease, previous to its erosion and destruction [46] and the synovial membrane revealed moderate to severe inflammatory changes; in contrast, probably because the study was conducted in an early model, the subchondral bone did not present the sclerosis typical to this disease. Thus, the results of the present study indicate that ACLT and partial medial meniscectomy in rabbits produce detectable OA changes, supporting the outcomes of previous studies [45] and therefore the effects of these compounds should be subjected to further studies.

In the histological assessment of OA changes, Mankin [47] or modified Mankin scoring systems in decalcified samples are considered as the gold standard [42]. However, because of its subjectivity and the possibility of inter- and/or intra-observer variability, the histomorphometry of calcified samples, using computer analysis systems, was introduced with a greater degree of objectivity and reproducibility [42].

Micro-CT has become in recent years the gold standard for three-dimensional analysis of bone microstructure, but when dealing with soft tissues, as cartilage, the technique usually has to be modified using complex staining methods with radiopaque contrast agents, due to its low X-ray transmission [48,49]; in the present study, the scanning conditions were adjusted to achieve a correct visualization of the cartilage, sufficient to provide any quantification of its morphology without staining (the scanning conditions used have been previously published [34]), thus the same biopsy could be used for both micro-CT and histomorphometry. The fact that PC-based histomorphometry and micro-CT techniques proved to be reproducible and objective methods is very significant.

In this study, for a complete characterization of cartilage, subchondral bone and synovial membrane in OA and the evaluation of the effects of different chondroprotective drugs, quantitative (histomorphometry) and qualitative microscopic evaluations were used, as well as micro-CT scanning. The results obtained by micro-CT and histomorphometry were comparable and better than those obtained for the decalcified samples, probably due to the combination of a better structure conservation of the calcified tissues (chiefly bone and calcified cartilage) that could be altered by decalcification [42] and the lack of possible substantial intra- and inter-observer variability of qualitative scoring methods.

Analyses of several randomized clinical trials and animal studies support the contention that the drugs studied are symptom-modifying agents, and may be structure-modifying agents, whereas others have detected no effect. These compounds are currently recommended by the EULAR (European League Against Rheumatism) [50,51] and the ESCEO (European Society for Clinical and Economic aspects of Osteoporosis and Osteoarthritis) [52] whereas, in contrast, in 2014 they were recommended as “uncertain” by the OARSI (Osteoarthritis Research Society International). The OARSI experts did not consider the term “uncertain” as a negative recommendation; rather it requires a role for physician-patient interaction because of their favorable risk-benefit ratio and its moderate to high effect size. In the case of risedronate, the last update of the OARSI consider it as not recommendable because of the lack of well-conducted studies to guarantee its efficacy [53].

In our study none of the treatments administered presented a remarkable better efficacy over the others; however, all of them, to a greater or lesser extent, demonstrated a tendency towards the amelioration of the pathological changes evaluated in cartilage, bone or synovial membrane. When the thickness and volume of the cartilage were measured (both micro-CT and histomorphometry) all the treatment groups achieve scores halfway between negative and positive controls, but without statistical differences. As mentioned before, several studies in animal models of early OA identified an initial phase of cartilage hypertrophy prior to its degeneration and loss [46] and, although the explanation of this phenomenon was not clarified yet, it could be an expression of the inflammation and an attempt of tissue reparation. In the present study the results confirm that with the administration of either of the studied compounds this related phenomenon was less pronounced, indicating its possible anti-inflammatory effect on cartilage.

The anti-inflammatory effect observed on the synovial membrane was different for the drugs used: diacerein and risedronate (alone or with glucosamine) presented a higher degree of effectivity as anti-inflammatories against synovitis, whereas glucosamine and chondroitin sulfate did not show any effect in ameliorating synovitis, and the hyaluronic acid was in-between the others; these results shown by glucosamine and chondroitin were different to previously published studies, in which improvement in the synovial inflammation was observed [54,55].

Regarding the superficial fibrillation index (FI) –an important parameter that provides a significant discrimination between ill and healthy animals [56]- the diacerein and the risedronate treatment (alone or combined) suggested a possible effect in slowing the disease corroborating the results previously observed by other authors [57,58]; whereas, the treatment with glucosamine alone or with chondroitin sulfate did not ameliorate the cartilage superficial fibrillation, which is in accordance with the results obtained in previous studies, where authors did not find prevention of the fibrillation or erosions of the articular cartilage surface with these drugs [8,59].

Finally, in this study the subchondral bone thickness and volume were not altered in any group; however, diacerein, hyaluronic acid and risedronate (alone and combined) seemed to considerably modify the orientation of the subchondral trabecular lattice (resented by a reduction in trabecular separation and an increase in trabecular number, a trend to bone formation as well as an increase in bone mineral density). Early in the pathogenesis of OA, prior to the development of sclerosis, there is a period of periarticular osteopenia [60] and a significant reduction in bone mineral density was reported in patients with mild OA when it was compared to healthy ones [61]. These effects on bone were expected in the animals treated with risedronate, a potent aminobisphophonate, which showed its effectiveness in conserving the periarticular bone [62]. In the same way, high molecular weight hyaluronic acid was shown to promote human osteoblast bone matrix protein expression *in vitro* [63] and bone formation *in vivo* [64] as well as to reduce bone resorption, but no study on this effect of diacerein on bones, to the best of the authors’ knowledge, has been published yet.

## Conclusions

The aim of the study was to compare the effects of the treatment with different OA drugs on an early model in rabbits. Out of the drugs used, diacerein and risedronate (alone or combined with glucosamine) were the compounds with a better profile of effectiveness in ameliorating these early changes, showing a better anti-inflamatory effect (in cartilage and synovial membrane), improving the cartilage surface alterations and increasing bone density. On the other hand, hyaluronic acid ameliorated cartilage swelling and promoted bone formation, but had less effect on the synovial membrane and its effects in preventing cartilage superficial erosion were also milder than those of risedronate and diacerein. Finally, in the present study, glucosamine and chondroitin sulfate only prevented cartilage swelling, in a similar way as the others, but had no effect on the cartilage surface, synovial membrane or subchondral bone.
